# Modeling of Acoustic Emission Signal Propagation in Waveguides

**DOI:** 10.3390/s150511805

**Published:** 2015-05-21

**Authors:** Andreea-Manuela Zelenyak, Marvin A. Hamstad, Markus G. R. Sause

**Affiliations:** 1 Institute for Physics, University of Augsburg, Universitätsstraße 1, Augsburg D-86159, Germany; E-Mail: markus.sause@physik.uni-augsburg.de; 2 Daniel Felix Ritchie School of Engineering and Computer Science, University of Denver, Denver, CO 80208, USA; E-Mail: Marvin.Hamstad@du.edu

**Keywords:** waveguide, acoustic emission, Lamb waves, propagation

## Abstract

Acoustic emission (AE) testing is a widely used nondestructive testing (NDT) method to investigate material failure. When environmental conditions are harmful for the operation of the sensors, waveguides are typically mounted in between the inspected structure and the sensor. Such waveguides can be built from different materials or have different designs in accordance with the experimental needs. All these variations can cause changes in the acoustic emission signals in terms of modal conversion, additional attenuation or shift in frequency content. A finite element method (FEM) was used to model acoustic emission signal propagation in an aluminum plate with an attached waveguide and was validated against experimental data. The geometry of the waveguide is systematically changed by varying the radius and height to investigate the influence on the detected signals. Different waveguide materials were implemented and change of material properties as function of temperature were taken into account. Development of the option of modeling different waveguide options replaces the time consuming and expensive trial and error alternative of experiments. Thus, the aim of this research has important implications for those who use waveguides for AE testing.

## 1. Introduction

Waveguides are used in acoustic emission (AE) detection to perform nondestructive testing (NDT) of structures under extreme operation conditions. For example, where high temperatures are such that the AE sensor with the active element made of piezoceramic lead-zirconate-titanate (PZT) cannot operate. The idea to mount piezoelectric sensors outside the extreme operation conditions came from researchers who used waveguides for nuclear applications [1]. Lynnworth *et al.* [2,3] used thin rods, clad rods or hollow tubes in between the piezoelectric sensor and the monitored high temperature structure. For example metallic buffer rods were used to monitor ultrasonic waves in molten aluminum at a temperature of 700 °C [4]. In the past, experimental studies were carried out to evaluate how close an AE signal detected by a sensor mounted on a waveguide duplicates the AE signal which was detected without the waveguide [5]. Both signals showed good agreement in terms of their shape but also highlighted the fact that the signal amplitudes reduces by 13 dB for a 1.59 mm aluminum waveguide of 306.4 mm length. The diameter of the waveguide had also an influence on signal degradation indicating a loss of duplication [5]. In practice, choosing the right waveguide to be used for measurements which involve temperatures outside the sensor specification, radiation, aggressive chemical environments or just objects much smaller than the sensor can be a real challenge. Typically, time consuming experiments must be performed before starting the measurement on the real test object, which can lead to high costs or destruction of AE sensors. Alternatively, the development of computational power and the high level of detail provided by FEM modeling, can lead to a simulation of AE signals propagation in waveguides is highly advantageous. In recent years various AE investigations on plate specimens were supported by simulations and their results compared to experimental data [6,7,8,9]. For AE signal detection PZT-based sensors are usually used. Using multiphysics FEM modeling platforms, like Comsol Multiphysics, allows including comprehensive AE sensor modeling. Recently, mass-backed piezoelectric sensor elements were numerically investigated using coupled physics between structural mechanics, piezoelectric effects and electrostatics in Comsol [7,10]. The approach presented in [7] includes a detailed analysis on a simple non-commercial system including full 3D-geoemtries and the interaction of the PZT-material with an attached circuit simulation.

The aim of this research is to investigate the waveguide (WG) influence upon AE signal propagation using numerical approaches taking into consideration the WGs radius and height. Different materials and material property changes due to the operating temperatures are included in the numerical investigations. The validation of the modeling approach was done by simulating the propagation of AE signals in a 3 mm aluminum plate with an attached stainless steel waveguide. The same configuration was also used to obtain experimental AE signals.

## 2. Simulation Methodology

The monitoring of guided waves in thin, plate-like structures is based mainly on the theory of plate waves, which are also known as Lamb waves [11,12,13]. In acoustic emission measurements of rather thin plates (e.g., the thickness of the plate being less than a few mm) the modes encountered most often are the fundamental symmetric mode S_0_ and the fundamental anti-symmetric mode A_0_.

**Figure 1:**
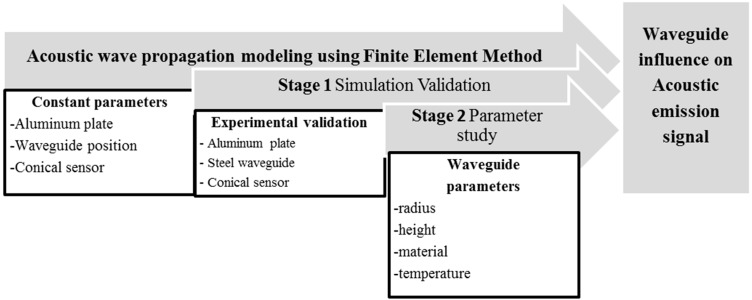
Overview of the present study.

Simulation of Lamb waves using the finite element method has already been achieved using different platforms [10,14,15]. In the finite element method the approach to simulate elastic waves is given by the time dependent solutions of partial differential equations of the equilibrium state [16]. For realistic modelling of experimental data, the acoustic emission sensor and the attached circuitry were also implemented in the model to include their interaction, which has been proven to have a strong impact on the AE signal [7]. Therefore, the foundation of the presented approach is based on previous investigations [5,17] and is now adjusted to the needs of the waveguide investigation. Our approach uses the Comsol FEM platform for coupling the structural mechanics module and the AC/DC module to model the guided wave propagation, the piezoelectric conversion and the attached circuit within a P-SPICE simulation. The present simulation work has two main stages which are presented in Figure 1.

The first stage is used to validate the simulation work through experimental results and at the second stage different waveguide parameters are changed and their effects are investigated.

### 2.1. Acoustic Emission Test Source

**Figure 2:**
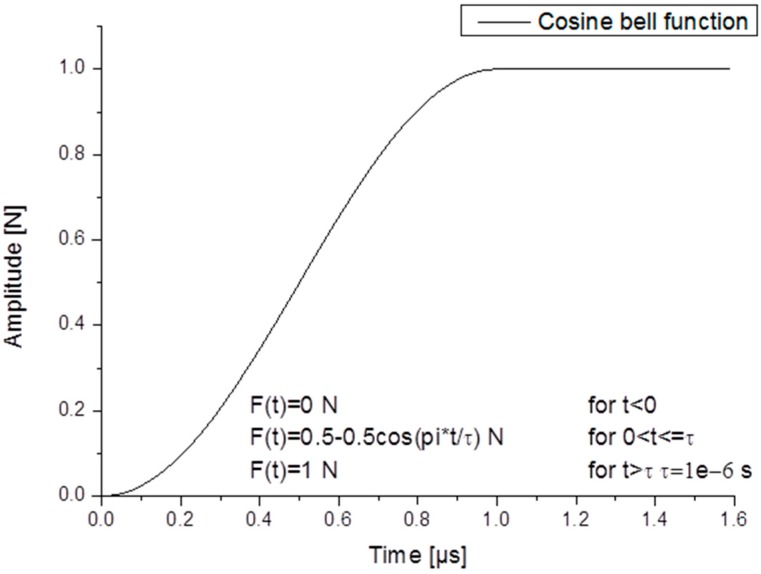
PLB‚ Cosine bell’ source characteristics.

As a typical test source used in literature, AE signals can be generated in plates using pencil-lead breaks (PLB) [5,18,19]. In this investigation the fundamental Lamb wave modes were excited using a monopole point source located on the top surface of the plate, acting in the out-of-plane direction. The temporal source characteristics can be seen in Figure 2a. It is called a “cosine bell” function selected to model the time dependence of the local surface deflection of the material due to a pencil-lead break. A maximum amplitude of 1 N was used for all simulation investigations except for the experimental validation of the FEM results when the maximum force amplitude was 3 N.

### 2.2. Geometry Setup

**Figure 3:**
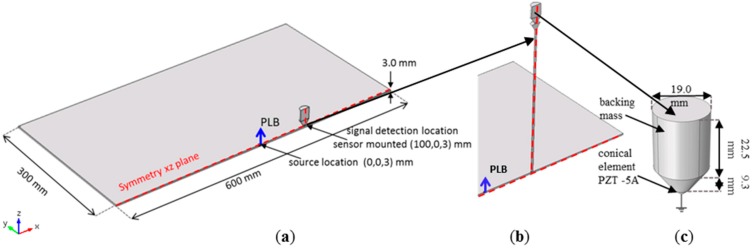
Geometry model for the (**a**) plate and sensor (**b**) plate, WG and sensor and (**c**) details of the conical sensor used [5].

The geometrical setup used for the simulations was chosen as close as possible to the experimental geometry applying some simplifications to reduce computational intensity. The 3-dimensional geometry used represents a 3 mm thick aluminum plate with 600 mm × 600 mm size having the AE sensor directly attached as seen in Figure 3a. The plate and the sensor were cut in the xz-plane to reduce the overall volume by a factor of two. Symmetry conditions were then chosen at the xz-plane. The PLB source is acting in the out-of-plane direction at the center of the plate. Previous investigations have shown that for this size of the plate no edge reflections occur at the detector position within 100 µs after signal excitation.

The sensor presented in Figure 3c is a conical type with a 1.5 mm aperture having the active element made of PZT 5A material. It is attached to the plate with a full solid contact as being welded. The sensor location was at 100 mm distance from the PLB source. This particular sensor model was chosen because of the geometrical simplicity making it easy to be implemented in the FEM simulation and because it was validated experimentally before [7]. All geometrical and numerical sensor characteristics were implemented as shown in Figure 3c and as presented in [7].

The waveguides were placed in between the plate and the sensor, at 100 mm distance from the source. One WG can be seen in Figure 3b. It is a WG with a conical termination on the upper end. Details about this particular waveguide will be given later in the description of the experimental setup. The symmetry condition at the xz-plane was also used for the models which involved usage of WGs.

## 3. Validation of Simulation

In this section the experimental setup used to validate the simulation work is described. Acoustic emission was generated using a PLB source on an aluminum plate. The detection was done using a conical element sensor directly applied on the plate and a second time with a waveguide in between.

### 3.1. Experimental Setup

**Figure 4:**
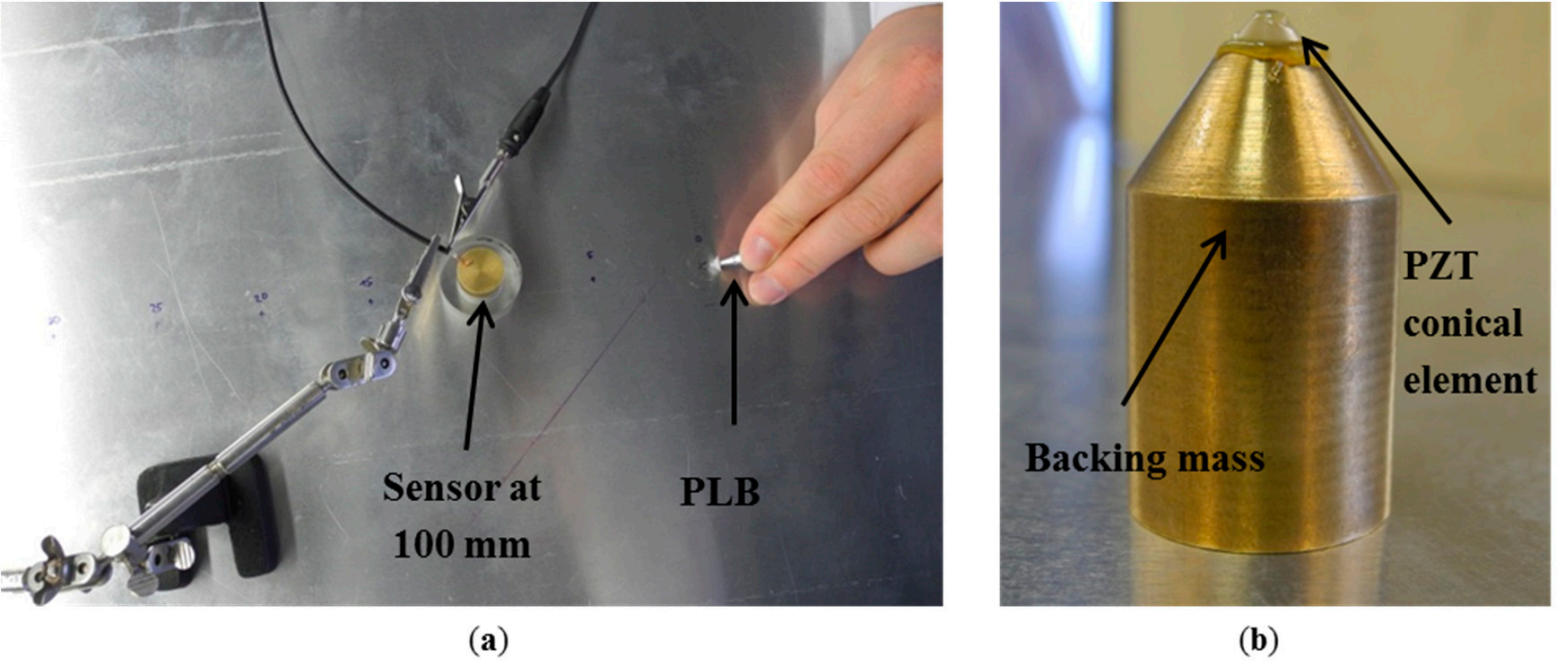
Photography of the experimental setup of the (**a**) plate and (**b**) details of the conical sensor.

The first experimental setup is shown in Figure 4a. Pencil-lead breaks using 0.3 mm diameter lead with 2H hardness and 3 mm length were applied on top of the aluminum plate. The plate dimensions of 2000 mm by 1000 mm wide and 3 mm thickness were large enough to avoid incident edge reflections at the sensor position for the duration of the direct signal.

The propagation distance was chosen to be 100 mm, distance which was large enough to allow the full development of the fundamental Lamb waves. This testing setup was used to obtain the reference AE signal. Details of the conical sensor are presented in Figure 4b. On top of the backing mass is the small piezoelectric PZT-5A active element with 1.5 mm aperture. Medium viscosity silicone grease was used to attach/couple the sensing element to the backing mass but also to couple the sensor to the plate. For the experiment an extremely thin layer of couplant was used to remove the air present due to the asperities on the two surfaces that are in “contact”. Thus with the couplant in place nearly perfect contact is present. For the modeled cases couplant is not required as the surfaces are perfect and do not have asperities. Thus perfect contact is present.

**Figure 5:**
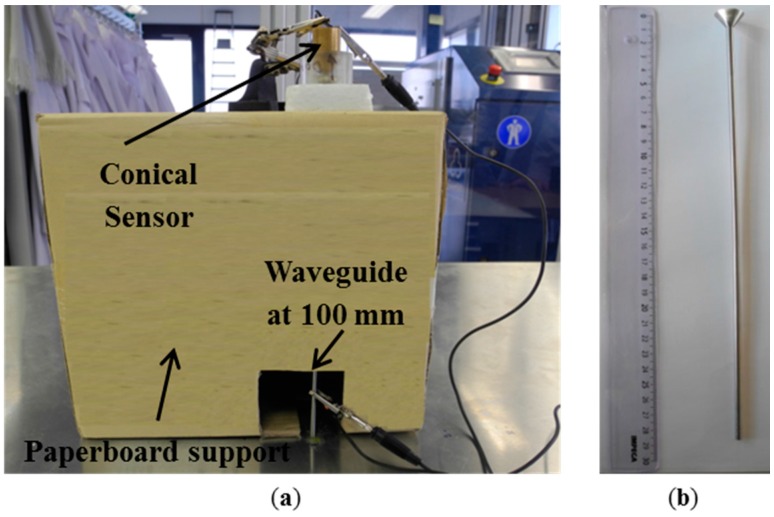
Photography of the experimental setup of (**a**) plate and waveguide and (**b**) photography of the conical waveguide.

For the second experimental investigation the conical waveguide was mounted perpendicular to the plate at 100 mm distance to the PLB position using low impedance materials as support structure. A detailed view of the setup can be seen in Figure 5a. The WG used had a circular cross section of 3.99 mm diameter and a length of 274 mm. The conical element attached to the upper part of the WG has a 32.55° semi angle and 17.62 mm diameter size at the upper surface with a total length of 10.68 mm. The same conical sensor as used in the previous investigation was mounted at the center of the upper surface of the WG in the same manner as in the plate case. A photograph of the waveguide used in the experimental measurements is presented in Figure 5b.

In both experimental configurations, the sensor was connected to a preamplifier with a total gain of 40 dB. All signals were acquired using threshold based signal triggering at 45 dB using a PCI-2 acquisition card with a band-pass filter ranging from 10 kHz to 3MHz. For each configuration ten consecutive pencil-lead breaks were recorded.

### 3.2. Simulation Details

Numerical convergence was achieved using 1 mm maximum element size for the mesh network along the wave propagation path and a distribution with maximum element growth rate of 1.05 for the entire plate. A maximum element size of 0.5 mm was used for the sensor. The types of elements used were tetrahedral using a Delaunay tessellation [20] for mesh growth. These mesh settings were chosen in accordance with other literature concerning AE wave propagation and were found to be convergent [8,9].

The selected time step was also an important factor when investigating the convergence of the numerical solution. From the numerical investigation conducted by Sause *et al.*, a 100 ns time step has shown good results having a coherence level of >0.99 [9]. A total duration of 150 µs was calculated for the model using a 100 ns time step without the waveguide and duration of about 250 µs for the investigation involving the conical waveguide. The reason for this extension of duration was to detect the signal on top of the waveguide. For the waveguide case, low reflecting boundary conditions were implemented at the boundary of the propagation domain in order to let the waves pass out without reflections from the edges. Simulation results were filtered with a 4th order Butterworth high-pass filter at 10 kHz to take into account the experimental band-pass filter.

For the aluminum plate, AlMg3 alloy properties were used, and for the waveguide those of steel were used. The elastic properties and electric properties of the materials assigned to the domains used in the modeling work are included in Table 1.

**Table 1:** Set of material constants used in simulation.

Property	AlMg_3_	Steel	PZT-5A	Brass UNS C2600	Alumina
Density [kg/m^3^]	2660	7850	7750	8627.6	3900
Elastic modulus [GPa]	70	200	C_11_ = C_22_ = 120.3; C_12_ = 75.2; C_13_ = C_23_ = 75.1; C_44_ = C_55_ = 21.1; C_66_ = 22.6	113.3	300
Poisson ratio	0.33	0.33	-	0.34	0.222
Rod sound velocity [m/s]	5129.89	5047.54	-	3623.84	8770.5
Piezoelectric constants [C/m^2^]	-	-	S_31_ = S_32_ = −5.4 S_33_ = 15.8 S_24_ = S_15_ = 12.3	-	-
Electrical permittivity	-	-	χ_11_ = 919.1 χ_22_ = 919.1 χ_33_ = 826.6	-	-

### 3.3. Comparison of Simulation vs. Experiment

**Figure 6:**
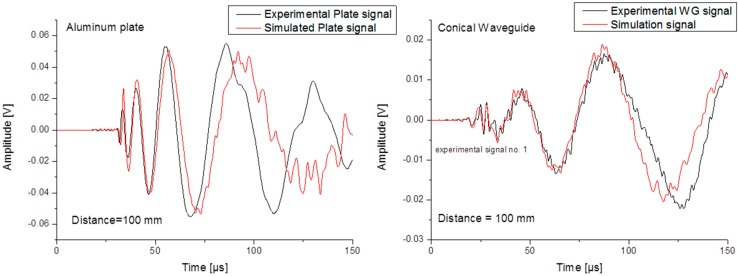
Experimental results *vs.* simulation results for (**a**) plate signal-reference (**b**) WG signal.

In this section the experimental signals are compared to the simulated signals in order to validate the modeling approach before expanding this numerical investigation to different materials, WG designs or operating conditions. Figure 6 shows the direct comparison between experimental signals and simulated signals.

The signals detected without the WG are showing the presence of the fundamental modes S_0_ and A_0_ in both cases. Only small discrepancies in magnitude are observed in the duration of 100 µs, which can be attributed to the attached circuitry for the cables and preamplifier, as previously shown in [7]. Beyond 100 µs duration of the simulated signal reflections from the domain edges can be observed contributing to the signal change in shape and amplitude. In comparison, the signals including the WG are looking almost the same. The amplitude of the signals is reduced for both S_0_ and A_0_ modes. For this case the difference between simulation and experiment may also be attributed to the attached circuitry but also to usage of pencil-lead breaks as test source which can induce strength variation in the source magnitude [21].

**Figure 7:**
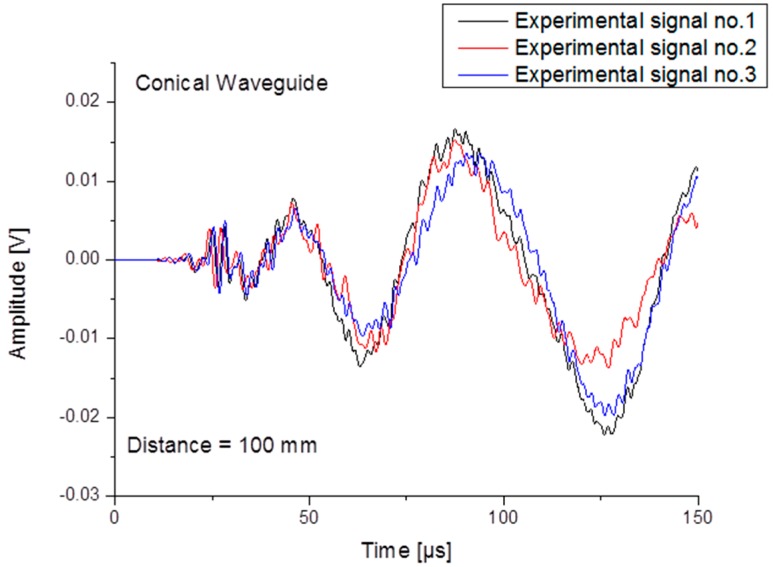
Experimental WG signals generated by different PLBs.

When comparing a set of different experimental signals generated by different PLBs, as presented in Figure 7 the variation of signal magnitude can readily be estimated. We can conclude that the numerical approach is feasible to describe the acoustic emission signal propagation for the simple plate configuration, but also when the configuration changes to a more complex setup like the conical shaped waveguide.

**Figure 8:**
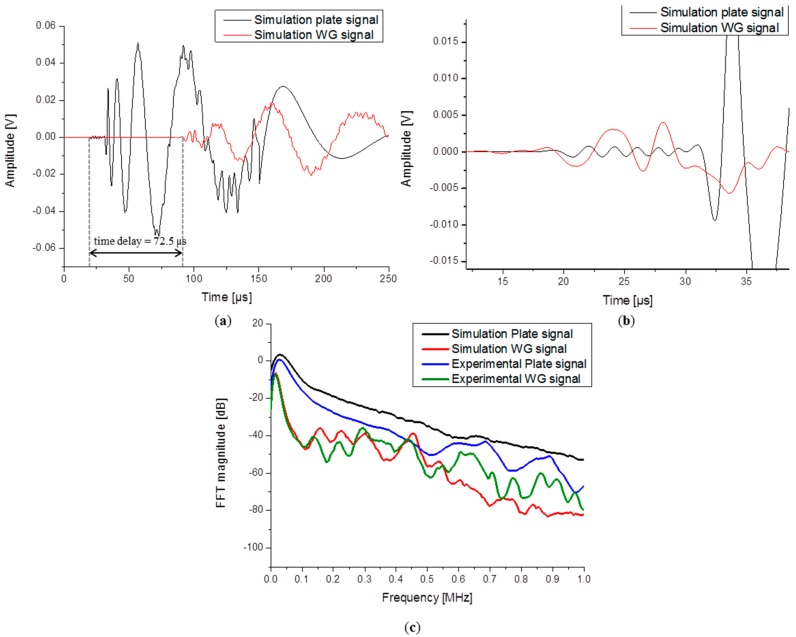
Comparison between (**a**) simulated signals and (**b**) simulation signals with a detail view of the S_0_ mode and (**c**) FFTs of simulated and experimental signals.

Time domain representations of simulated signals are shown in Figure 8a. An offset of 72.5 µs was found between the arrival time of the reference signal on the plate and the signal on the waveguide. The exact same value was also found for the experimental signals. This good agreement between the experimental and modeled signals arrival times especially validates the chosen material properties of Table 1.

As generally expected, the waveguide signal is lower in amplitude *vs.* the signal detected directly on the plate. The reduced amplitude detected by the sensor mounted on the waveguide can be attributed to the effect of WG diameter change but also to the loss of higher frequencies of the S_0_ and A_0_ modes which are not properly transferred to the WG as shown in previous experimental investigations [5]. A detailed view of the S_0_ mode is shown in Figure 8b. Careful inspection of the time domain signals reveals that not all modes are attenuated equivalently. The S_0_ mode detected on top of the WG exhibits an increased amplitude when compared with the reference signal. This can be due to the larger aperture of the waveguide in comparison to the sensor aperture. This tendency was pointed out before by Sause *et al.*, in the investigation concerning acoustic sensor modeling [7].

In order to demonstrate the signal attenuation due to the initial transmission from the plate and by traveling through the waveguide all results were transformed into the frequency domain using the Fast Fourier Transformation (FFT). The frequency spectra of the respective signals are shown in Figure 8c. The magnitude is compared in the low frequency range between 0 and 100 kHz. The overall magnitude difference was found to be 10 dB between the reference signals and the signals detected on the waveguide.

## 4. Parameter Study

### 4.1. Influence of Waveguide Geometry and Material

**Figure 9:**
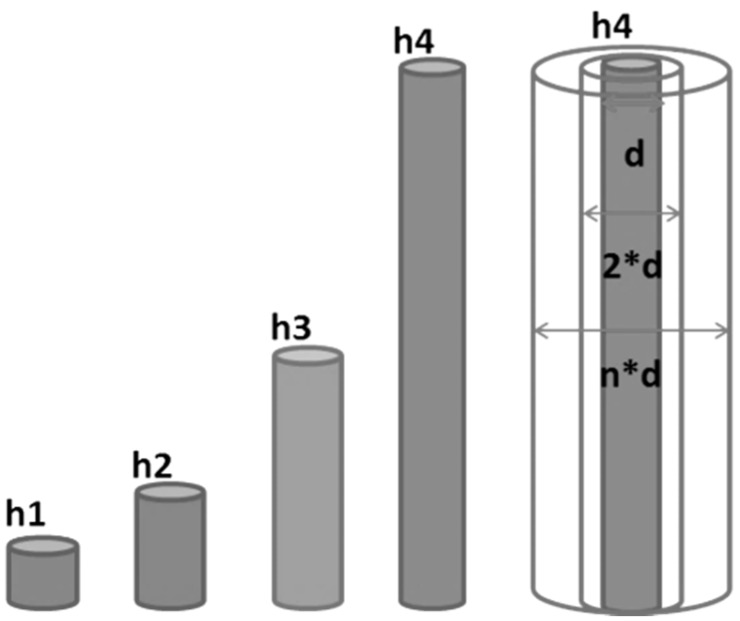
Schematic geometry of the waveguide cases studied.

The conical shaped WG geometry described in the previous section was primarily used for the validation purpose to comply with the experimental setup. For all other simulation studies a simple WG with constant circular cross section was used as shown in Figure 9. The geometrical impact upon the AE propagation was studied by changes in the geometry and radius of the WG as can be seen in Figure 9.

Four different WG lengths were implemented ranging from 76.5 mm to 306.3 mm. The diameter was changed from 0.795 mm to 7.950 mm in eight steps. Details of all considered values are reported in Table 2.

**Table 2:** Parameters for cases studies.

Parameters	Constant Values	Varied Values							
Length variation [mm]	Diameter = 1.59	76.57	153.15	229.71	306.3				
Diameter variation [mm]	Length = 306.3	0.79	1.59	2.38	3.18	3.97	4.77	5.56	7.9
Material variation	Diameter = 1.59 Length = 306.3	Aluminum		Steel		Alumina		Brass	

Acoustic signal transmission can also be affected by the WG material. Different types of materials were considered. For all cases the plate was made of aluminum and only the WG materials were varied as presented in Table 2. For all material variations the waveguide size was kept identical to assess only the changes of the AE signal due to the different materials.

All calculated signals were subject to the same post processing steps in order to have comparable signals. The steps follow the ones used by Hamstad *et al.*, in his experimental study regarding small diameter waveguide for wideband acoustic emission [5], which were also used by the authors in a previous investigation [17]. Keeping the same data processing allows comparing this work with the previous findings in a direct way. The data processing steps used are:

- The WG signal was shifted forward in time to superimpose
- All signals were terminated at a convenient zero
- All signals were extended with 0 values to a total length of 2048 points
- The Fast Fourier Transformation (FFT) was calculated with a square window function
- The resulting FFTs were smoothed by a 30 point Savitzky-Golay (5th polynomial) method
- The FFTs magnitudes were compared from 0 to 100 kHz.

#### 4.1.1. Influence of WG Diameter

**Figure 10:**
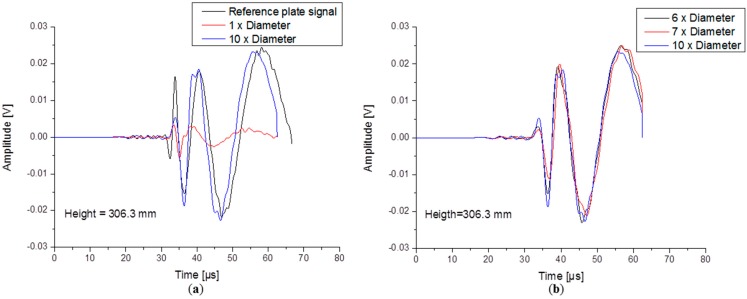
Time domain signals of the (**a**) extreme WG diameter cases and (**b**) intermediate WG diameter cases.

As in the validation step, the reference signal considered here is the one detected directly on plate. All the signals detected on top of the waveguides are considered to carry the “waveguide signature”. In Figure 10 the time domain representation of the calculated signals are directly compared. First, only the extreme cases were chosen to be plotted. The first signal plotted with red colour corresponds to the WG with 0.794 mm diameter and the blue one is given by the model with a ten times larger waveguide diameter. Those two cases are directly compared with the reference signal (black). The detected WG signals are clearly showing the presence of both fundamental A_0_ and S_0_ modes, duplicating the shape of the signal which was initially propagating in the plate. In the smallest rod case (0.795 mm diameter), the duplicated WG sensor signal is very small in amplitude in comparison to the reference signal. In contrast, the larger diameter WG transmits a signal of almost the same amplitude and shape particularly in the A0 region. In Figure 10b signals generated by the models with waveguides having diameter dimensions in between the two extreme cases are presented.

**Figure 11:**
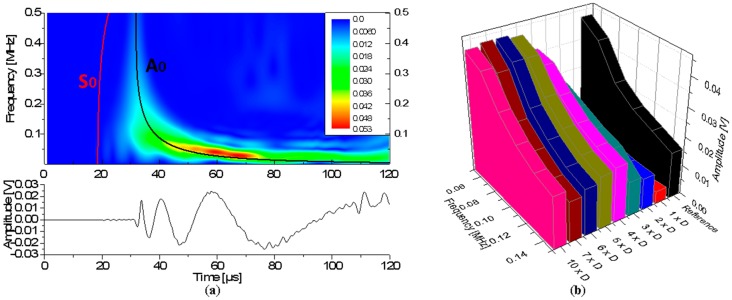
(**a**) Wavelet transform of reference case and (**b**) maximum A_0_ amplitude as function of WG diameter and frequency.

Signals generated by all diameter cases were subject to a Wavelet Transformation (WT) and their wavelet coefficient intensity was used to extract the maximum amplitude at different frequencies. The WT was performed using the AGU Vallen software, with a WT diagram of the reference signal shown in Figure 11a.

In order to compare the signal changes induced by the WG, the maximum magnitude of both fundamental modes was evaluated at constant frequency for each WG diameter case. Overall the signal amplitude has a tendency to increase with higher diameter WGs.

**Figure 12:**
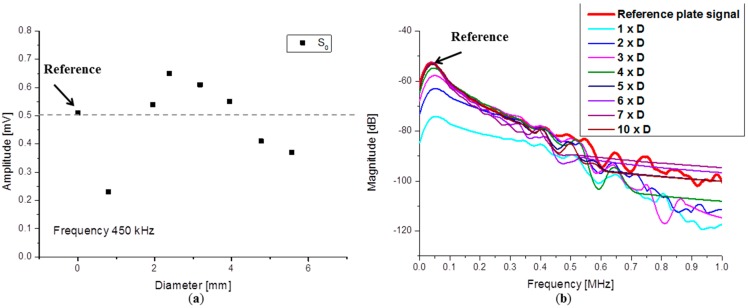
Magnitude (**a**) of S_0_ mode from WT (**b**) from FFT.

Such a comparison can be seen in Figure 11b for the A_0_ mode and in Figure 12a for the S_0_ mode. The basic conclusion here is that the A_0_ amplitude is increasing with the WG diameter but the transmitted signal amplitude tends to saturate when the WG diameter dimensions approaches the value of 4.77 mm. The change in signal amplitude is a consequence of diameter variation of the WG, a finding which was found to be valid for a broad frequency range.

The same behavior is found for the S_0_ mode. The detected S_0_ mode amplitude is almost the same as in the reference case when a larger waveguide diameter was used. However, a lower WG diameter size seems to be already sufficient to achieve the same amplitude (Figure 12a). This corresponds to a waveguide with the diameter of 1.59 mm, a size which is close to the PZT sensor tip diameter of 1.5 mm. Increasing waveguide diameters yield an increase of the S_0_ mode amplitude until it saturates and finally starts to decrease. From the FFT magnitudes (Figure 12b) it can also be seen that the highest magnitude offset of 22 dB is induced by the usage of the smallest diameter waveguide.

#### 4.1.2. Influence of Waveguide Length

**Figure 13:**
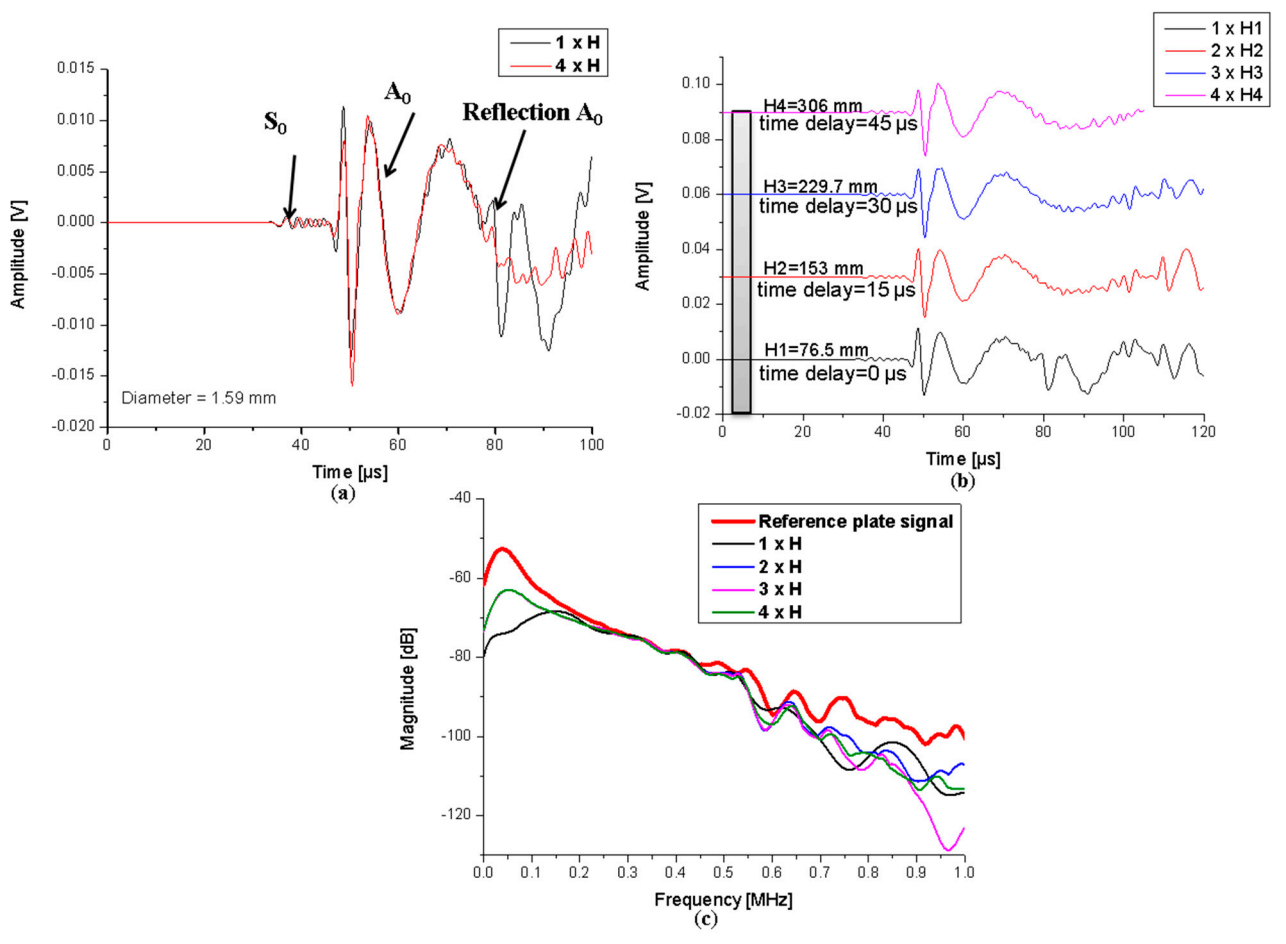
Calculated signals for (**a**) extreme WG length cases; (**b**) all cases and (**c**) frequency spectra thereof.

Waveforms of the simulations using waveguides with extreme lengths are presented in Figure 13a. From the time domain representation of the signals it can be seen that both fundamental modes are present and the wave shape is not affected much by the WG length. In the shortest WG case (height of 76.57 mm) multiple reflections (from the ends of the WG) of the modes are detected, which are not present in the longest WG case. All signals computed for the four different WG lengths are presented in Figure 13b. Reflections can be observed to vanish for all increasing lengths of the WGs. All signals were subject to FFT and analyzed with respect to their magnitude.

The frequency spectra presented in Figure 13c show that the signals from 0 to 100 kHz are reduced in magnitude by 10 dB, with exception being made by the shortest waveguide for which the attenuation is even higher at 20 dB. The latter effect is attributed to the multiple reflections which occur during the signal time. A time delay of 15 µs induced by each waveguide in arrival time is noticed, which can be associated with the 76.5 mm extra height added to each case.

Overall the FFTs of the signals detected with waveguides of different lengths are not providing substantial deviations to the reference case and no significant magnitude reduction when compared relative to each other. Thus for the given range of investigation, the choice of the waveguide length does not seem to be of vital importance and thus may be adapted to the experimental needs. These may originate from the environmental conditions requiring the sensor to be located as far as possible from a harmful position.

#### 4.1.3. Influence of Waveguide Material

In practice, waveguides are made from different materials, which can have an impact on transmission of the signal or on the signal arrival time. In this section four types of waveguides are analyzed: metallic WGs made of aluminum, steel, brass and one ceramic WG made of alumina (cf. Table 1). All simulated waveguides are of the same diameter (1.59 mm) and length (306.3 mm). Comparison is made in the time domain representation of the signals to identify shape changes, shifts of arrival times or modal conversions and also using frequency spectra to compare the magnitude changes as function frequency. All other model parameters were kept identical to the reference case.

**Figure 14:**
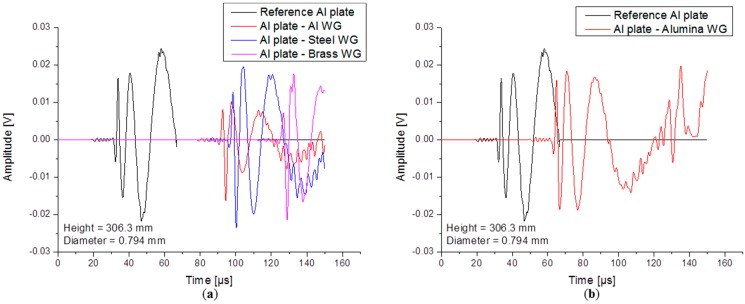
Calculated signals for (**a**) metallic waveguides and (**b**) ceramic waveguide.

In Figure 14 simulated signals of the waveguides are presented in comparison with the reference signal. The time delay between signals was not eliminated in order to visualize the material influence. The reference case shows the arrivals of the fundamental modes after 20.5 µs for S_0_ and 32.4 µs for A_0_. The aluminum WG induces a delay of 60.2 µs in arrival time, the steel WG 66.84 µs and the brass WG imposes an arrival time difference of 97.98 µs. In Figure 14b a comparison between the reference signal and the signal obtained on top of the ceramic WG is made. The difference in arrival time of 32.94 µs is much smaller in this case due to the high sound velocity of the alumina. For the same reason a first reflection of the modes is detected within the 150 µs duration. The presence of the S_0_ and A_0_ modes is clearly observed in all signals and no conversion of the modes was noticed.

**Figure 15:**
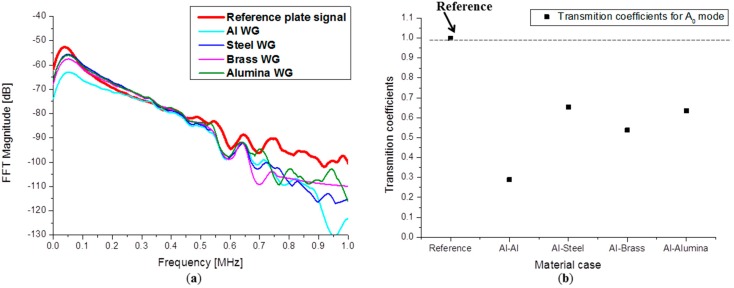
(**a**) Calculated FFT spectra and (**b**) A_0_ transmission coefficients.

Choosing an appropriate waveguide material can be a solution to obtain higher amplitudes. Signal transmission coefficients depend on the acoustic impedance of the materials forming the interface. The FFTs presented in Figure 15a show similar frequency spectra in the frequency range up to 1 MHz, but a clear difference in magnitude is noticed when compared to the reference signal or when compared relative to each other.

The largest decrease in magnitude is observed when using an aluminum waveguide with a total decrease of about 10 dB. Less amplitude reduction was observed in the case of a Steel WG and the alumina WG, both being about 3.5 dB. The transmission coefficients for the brass waveguide are somewhere in between steel and aluminum, but the signal detected in this case has 5 dB less amplitude than the reference signal. Transmission coefficients can be calculated when knowing the incident wave amplitude and the amplitude of the wave traveling into the waveguide. In Figure 15b a graphical representation of the calculated transmission coefficients for the A_0_ mode are plotted for all material combinations. The signal amplitudes were extracted using the maximum magnitude of the signals WT at constant frequency of 50 kHz. The highest signal transmission is obviously achieved when using steel and alumina waveguides.

### 4.2. Influence of Waveguide Temperature

Changes in waveguides due to temperature are an important factor when considering the usage of waveguides in acoustic emission measurements. Typical questions of sufficient waveguide length in order to avoid harmful temperatures at the sensor position arise when conducting measurements in such extreme temperature conditions. In this section, the presented model is extended by a coupled heat transfer analysis. This was implemented in Comsol Multiphysics by adding the Heat Transfer module to the previous modules. A two-steps study was used for the computation. The first study step was considered to be stationary and was used to solve the heat transfer equations assuming that the temperature is in a stationary state during the moment of signal propagation. The temperature distribution inside the waveguide was obtained by two temperature boundary conditions. At the plate all boundaries were set to 520 °C and on the upper end of the waveguide, the boundary was set at 20 °C temperature.

**Figure 16:**
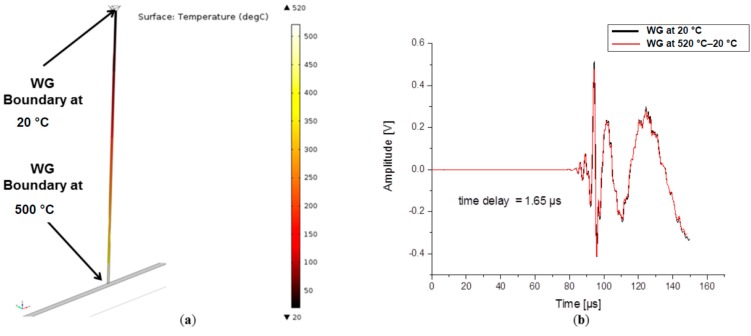
(**a**) Temperature distribution in plate and waveguide and (**b**) comparison of signals at room temperature and with elevated temperature.

The obtained temperature distribution after the stationary study step can be seen in Figure 16a. In the second study step, the model was solved as previously described, but with the superimposed temperature distribution and temperature dependent material properties as listed in Table 3. The WG and plate material used for this simulation was the iron alloy UNS K12211. The waveguide used had a circular cross section with a diameter of 1.59 mm and a length of 306.3 mm.

To compare the results and to see the impact of the temperature on signal propagation, a second model with all components at room temperature of 20 °C was used as reference. All calculations were performed for 160 µs duration. The time domain signals in Figure 16b show two signals which are dominated by the A_0_ mode with no significant difference in shape and amplitude. A time delay of 1.65 µs in arrival time is noticed, which can be directly related to the change of material properties and geometric expansion due to the temperature difference.

In summary, the overall appearance of the signals in Figure 16b leads to the conclusion that temperature difference of 500 °C has almost no effect on the signal shape and amplitude as long as the material properties change only for the same amount as the iron alloy UNS K12211 investigated in the present case.

**Table 3:** Temperature-dependent material properties.

Property	UNS K12211
Density [kg/m^3^]	$7919.309-0.124948*T^{1}-2.88651\;10^{-4}*T^{2}+1.131694\;10^{-7}*T^{3}$ for 293K<T≤1605K
Elastic modulus [GPa]	$2.109875\;10^{11}+3.572844\;10^{7}*T^{1}-106319.6*T^{2}$ for 293K<T≤1605K
Poisson ratio	$0.2712267+7.030261E\;10^{-5}*T^{1}-3.856929\;\;10^{-8}*T^{2}+1.246582\;10^{-11}*T^{3}$ for 273K<T≤1053K
Thermal conductivity [W/m*K]	$1.842649\;10^{11}-2.509462\;10^{7}*T^{1}-28588.37*T^{2}$ for 273K<T≤1500K
Heat capacity at constant pressure [J/kg*K]	$-215.7306+6.0185*T^{1}-0.01834293*T^{2}+2.414973\;10^{-5}*T^{3}-1.078824\;10^{-8}*T^{4}$ for 293K<T≤848K

## 5. Conclusions

In this paper it has been shown that finite element modeling can be used to simulate acoustic emission wave propagation generated by pencil-lead breaks in thin plates and rods. Experimental work conducted in this research has been used to validate the presented simulation approach.

Extended numerical investigations have been used to evaluate the influence of the waveguides geometry and material on signal propagation. It has been demonstrated that the waveguide diameter will predominantly affect the signal amplitude. For example an aluminum waveguide with 4.77 mm diameter allows reaching the same signal A_0_ mode amplitude as a direct detection of the signal on the plate. The waveguide length did not show a significant influence on the detected signals as far as their frequency spectra is concerned but may lead to early reflections if chosen too short. Acoustic impedance mismatches were analyzed by changing the waveguide materials. Steel or alumina waveguides were found to improve the detection of signals in an aluminum plate by 65% in terms of magnitude when compared with an aluminum waveguide. Also the impact of the waveguide material may be considered for further investigations, when the signal arrival time may have an impact on the sensor operation or possibly destruction if it is exposed to extreme environmental conditions. The numerical investigation concerning the influence of elevated temperatures on acoustic signal propagation has demonstrated the possibilities of a coupled multiphysics analysis. It could be observed, that no significant changes of the signal occur, despite of the 500 °C temperature difference between the plate and the end of the waveguide (detection point). This work emphasizes the possibility of modern computational methods to replace some experimental efforts by skilled numerical analysis using FEM methods to establish a suitable type of waveguide for an indirect acoustic emission measurement.
